# Basic life support knowledge of first-year university students from Brazil

**DOI:** 10.1590/1414-431X20154667

**Published:** 2015-09-18

**Authors:** S. V. Santos, M. R. R. A. Margarido, I. S. Caires, R. A. N. Santos, S. G. Souza, J. M. A. Souza, R. R. Martimiano, C. S. K. Dutra, P. Palha, A. C. G. Zanetti, A. Pazin-Filho

**Affiliations:** 1 Programa de Educação Tutorial, Faculdade de Medicina de Ribeirão Preto, Universidade de São Paulo, Ribeirão Preto, SP, Brasil; 2 Programa de Educação Tutorial, Escola de Enfermagem de Ribeirão Preto, Universidade de São Paulo, Ribeirão Preto, SP, Brasil

**Keywords:** Basic life support, Undergraduate education, Cardiac arrest, Pre-hospital, First Aid

## Abstract

We aimed to evaluate knowledge of first aid among new undergraduates and whether it is affected by their chosen course. A questionnaire was developed to assess knowledge of how to activate the Mobile Emergency Attendance Service - MEAS (Serviço de Atendimento Móvel de Urgência; SAMU), recognize a pre-hospital emergency situation and the first aid required for cardiac arrest. The students were also asked about enrolling in a first aid course. Responses were received from 1038 of 1365 (76.04%) new undergraduates. The questionnaires were completed in a 2-week period 1 month after the beginning of classes. Of the 1038 respondents (59.5% studying biological sciences, 11.6% physical sciences, and 28.6% humanities), 58.5% knew how to activate the MEAS/SAMU (54.3% non-biological *vs* 61.4% biological, P=0.02), with an odds ratio (OR)=1.39 (95%CI=1.07-1.81) regardless of age, sex, origin, having a previous degree or having a relative with cardiac disease. The majority could distinguish emergency from non-emergency situations. When faced with a possible cardiac arrest, 17.7% of the students would perform chest compressions (15.5% non-biological *vs* 19.1% biological first-year university students, P=0.16) and 65.2% would enroll in a first aid course (51.1% non-biological *vs* 74.7% biological, P<0.01), with an OR=2.61 (95%CI=1.98-3.44) adjusted for the same confounders. Even though a high percentage of the students recognized emergency situations, a significant proportion did not know the MEAS/SAMU number and only a minority had sufficient basic life support skills to help with cardiac arrest. A significant proportion would not enroll in a first aid course. Biological first-year university students were more prone to enroll in a basic life support course.

## Introduction

First aid courses are common in developed countries, but much less so in developing countries such as Brazil. We have recently demonstrated the feasibility of including cardiopulmonary resuscitation (CPR) courses as part of early undergraduate education in Brazil, but we are not certain if these students have any contact with first aid information before admission to a university (1). If they do, most of it would probably be provided informally, such as via television, the Internet, or individual research projects (1-3).

Less than 10% of the Brazilian population has access to a higher education. This group is therefore an elite population that could have studied first aid as part of a basic education in preparation for the Brazilian university admission evaluation. University preparatory courses do not provide this kind of information.

Recognizing emergency situations and being able to activate the Mobile Emergency Attendance Service - MEAS (Serviço de Atendimento Móvel de Urgência - SAMU) are basic abilities that every citizen should have. This elite group of university students will be future leaders, and therefore have influence. We therefore sought to evaluate the degree of knowledge in this specific population. This may have an impact on the structure of early undergraduate teaching.

## Material and Methods

A dedicated questionnaire was developed to assess students’ knowledge of how to activate MEAS/SAMU), recognize a pre-hospital emergency situation and perform first aid for cardiac arrest. The questionnaire had two sections; the first asked about personal demographic characteristics (age, sex, place of birth, previous university degrees, and family history of heart problems) and about their interest in taking a first aid course.

The second section consisted of three questions. To evaluate the first objective, we used a simple question asking respondents to write down the MEAS/SAMU telephone number (192). This would give a better answer than binary or multiple choice questions, avoiding bias. For the second question, we chose four common conditions, two of which were clearly defined emergencies (acute coronary syndrome and stroke), which would require the activation of the MEAS/SAMU, while the other two (hangover and indigestion) would not. We did not include a trauma scenario because the presence of blood is culturally considered to be an emergency regardless of severity. The situation was described and the subject was asked to answer yes or no for each one. Blank questions were considered as “No” for analytical purposes. The third question was open-ended, with a short description of a cardiac arrest scenario, and the subject was asked what actions they would take if faced with this situation. Activating the pre-hospital emergency system was not considered in this question, since the two previous questions could have biased the response. We considered that the two actions required of laypeople providing basic life support would be external cardiac massage (chest compressions) and use of an external automated defibrillator (EAD) (4,5).

The primary researchers developing the questions were all students in medicine and nursing courses. They, therefore, used appropriate language for the students’ age, while avoiding slang. The senior researchers provided input to the question development, which improved the questionnaire. A final version was administered at the end of 2012 to 15 first-year undergraduate students studying two courses in each broad area (biological sciences, physical sciences, and humanities). No problem was detected with the students’ comprehension and this final version of the questionnaire was subsequently used more widely (6).

We did not use sampling methods because we tried to reach every student. The questionnaire was administered to 1038 of the 1365 (76.04%) new undergraduates of the colleges of the Campus de Ribeirão Preto, Universidade de São Paulo, within a 2-week period, 1 month after the beginning of the 2013 academic year. We chose this period to avoid any contamination from the content of university courses, since no course included a basic life support element prior to this time. The questionnaire was administered by the researchers at the beginning of one class in each course. We included only those who answered the questionnaire in person, while the researchers were present. No digital form was used, and no questionnaires completed after the researchers had left the class were included in the analysis. The questionnaire application time was about 5 to 10 minutes for each session and all the instructions were contained within the questionnaire. There was no intervention from the researchers other than time control.

After collecting the information, we derived binary variables for analysis. The only continuous variable used was age. The means±SD or percentages were applied as central tendency measures, depending on the nature of the variable. A univariate analysis was performed with a Student’s *t*-test or chi-square test as appropriate. A multivariate analysis was performed using logistic regression. We constructed forward regression models, parting from the outcome and the group until we had established the final model. This strategy was used to help evaluate the presence of collinearity. All analysis and graphic generation was performed using STATA version 10 (Statacorp, USA).

The study was approved by the Ethics Committee of Hospital das Clínicas, Faculdade de Medicina de Ribeirão Preto, Universidade de São Paulo. All participants signed a written consent form prior to enrollment.

## Results

Of 1365 first-year students, 1038 (76.04%) completed the questionnaire. A total of 30 cases were lost because identification was compromised, so the final study population was 1008 students, with 290 (28.7%) studying humanities, 117 (11.6%) physical sciences, and 601 (59.6%) biological sciences. Because the majority of students on the campus study biological sciences, we decided to group the humanities and physical sciences courses for analytical purposes, calling them non-biological (NBio) as opposed to biological sciences (Bio).

The only demographic difference between the two groups was the proportion of males (NBio 52.3% *vs* Bio 31.8%, P<0.01) although there was a slight difference in the proportion living in Ribeirão Preto prior to admission (NBio 32.2% *vs* Bio 26.6%, P=0.056). There was no difference in age in years (mean±SD; NBio 19.5±3.8 *vs*18.9±2.1), being a graduate of another university course (NBio 13.5% *vs* 10.3%) or having a relative with heart disease (NBio 45.2% *vs* Bio 45.5%).

**Figure 1:**
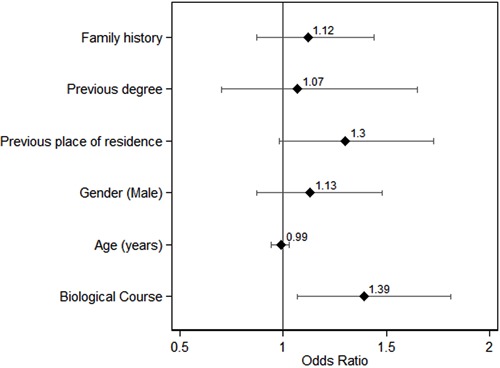
Odds ratio and 95% confidence interval among first-year undergraduates at the University of São Paulo Ribeirão Preto Campus (2013 academic year) for providing the correct number for the Mobile Emergency Attendance Service - MEAS (Serviço de Atendimento Móvel de Urgência; SAMU).

Of the students, 58.5% would be able to activate the MEAS/SAMU (Table 1). When separated by subject, the success rate was 61.4% for those studying biological sciences and 54.3% for other subjects (P=0.02), with an odds ratio (OR)=1.39 (95%CI=1.07-1.81) regardless of age, gender, origin, having a previous degree, or having a relative with cardiac disease (Figure 1). The majority of the students were able to differentiate emergency (heart attack/stroke) from non-emergency situations (hangover/indigestion; Table 1).

**Table:**
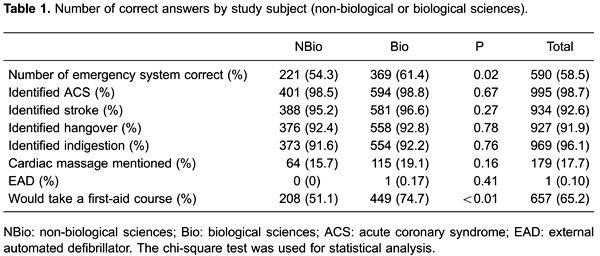

**Figure 2:**
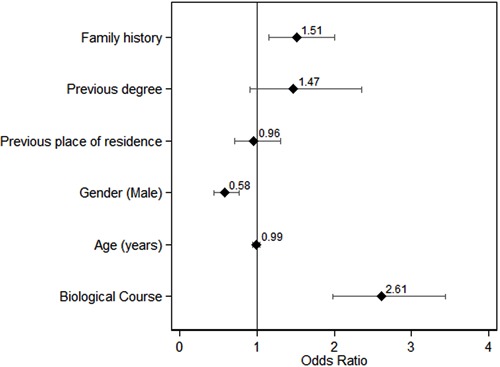
Odds ratios and 95% confidence intervals for mentioning cardiac massage among first-year undergraduates at the University of São Paulo Ribeirão Preto Campus (2013 academic year).

When faced with possible cardiac arrest, 17.7% of the students would perform CPR, irrespective of the group (NBio 15.7% *vs* Bio 19.1%, P=0.16). Only 3 people described the massage technique, 2 mentioned the frequency of the massage and 1 mentioned the use of an EAD; all of these were studying biological sciences. Studying biological sciences (OR=1.42; 95%CI=1.02-2.02), being male (OR=1.48; 95%CI=1.06-2.07) and having a relative with heart disease (OR=1.46; 95%CI=1.05-2.02) were independently associated with mentioning CPR in a multivariate logistic regression model (Figure 2).

**Figure 3:**
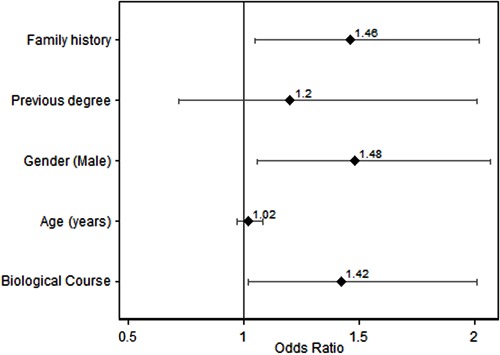
Odds ratios and 95% confidence intervals for interest in taking a first aid course among first-year undergraduates at the University of São Paulo Ribeirão Preto Campus (2013 academic year).

A total of 657 (65.2%) of the students would enroll in a basic life support course, with a significant difference between groups (NBio 51.1% *vs* Bio 74.7%, P<0.01). In the logistic regression final model (same variables adjusted as in the final model for activating the emergency system), studying biological sciences would result in an OR=2.61 (95%CI=1.98-3.44). The only other significant variable included in this model was being male, with an OR=0.58 (95%CI=0.44-0.77; Figure 3).

Finally, because the majority of the courses at this university are biological, we tried to evaluate the nature of the biological course, stratifying into two subgroups of Medicine and Nursing, and all other courses. No difference was observed between these subgroups.

## Discussion

We have demonstrated that although a high percentage of new undergraduates at a public university were able to recognize emergency situations, a significant fraction did not know the MEAS/SAMU phone number and only a minority would have the necessary basic life support knowledge to help with cardiac arrest. Even knowing this, a significant minority would not be interested in taking a basic life support course. The biological sciences students were more likely to know, and want to know, about basic life support.

Even though basic life support skills are considered mandatory in all health professions, it is very difficult to ensure this is the case (7). A nationwide policy may be required to achieve this, as in developed countries (8). Recent data from South Korea emphasized the importance of this type of support (9). Universities generally have younger populations than society as a whole, but cardiac arrest could still be an issue, because of the large number of people, elevated turnover and level of sports participation. Although rare, cardiac arrest in athletes is the primary cause of mortality among university students (10).

Knowing how to activate the pre-hospital emergency system is a fundamental element of basic life support (11). It is therefore very disturbing to find that almost 42.5% of an intellectual elite were not able to recall how to do so. Even though we did not ask the reason for not knowing, one possibility is that basic life support training is not provided before university study. The experience of our group in teaching basic life support to first year undergraduates seems to support this hypothesis (1). If this hypothesis is correct, the students may be acquiring some knowledge informally, for example, from television (2). Information acquired in this way could be misleading, however, some television programs are foreign, and provide, for example, the United States pre-hospital emergency number (911) instead of the Brazilian one (192). In practice, 5% of those who did not write down the correct MEAS/SAMU number answered 911, despite the MEAS/SAMU number being clearly marked on every public ambulance.

Identifying a possible emergency situation is another key element of basic life support. We tried to evaluate this with four scenarios that should be familiar to the population being studied. Most students had no problem identifying the emergencies. This may be because these topics are covered in schools. This is particularly so for acute coronary syndrome and stroke, for which there are huge campaigns by medical associations (12). Quite a large proportion of the students have or had a relative with heart problems, reflecting the prevalence of these conditions in the general population. We cannot rule out the questions being too easy, but our intention was to see whether students would recognize common scenarios.

Finally, being able to provide basic life support until advanced life support units arrive at the scene is an essential skill. We tried to evaluate that with a simple cardiac arrest scenario and an open-ended question. It was alarming that only 17.7% of the students mentioned cardiac massage, only three mentioned the required technique or frequency of compressions and only one mentioned using an EAD. This finding implies a lack of knowledge that should have been provided before this stage. As we have pointed out, this is not the reality in Brazil and there has been much discussion about how to educate and train the general population (5,13,14). Perhaps including a basic life support course as part of the university curriculum, regardless of the area of study, would help to disseminate this knowledge among the future leaders of society. Currently, first aid or basic life support courses are only provided as part of biological science courses. This suggestion is not new; it has been discussed in the literature for more than 15 years, including recently, but has not been explored or implemented. This again emphasizes the importance of policies on university curricula and public health (15-18).

Mentioning CPR in the open-ended questionnaire was positively associated with studying biological sciences, being male and having a relative with heart disease. These findings could partially explain why being male was inversely associated with interest in taking a first aid course. It is possible that those who already had some knowledge of first aid were less interested in taking a basic life support course. This knowledge, however, does not seem to be complete or sufficient, since there was no mention of the correct technique for CPR and the use of an EAD.

One of the major strengths of our work is the high coverage of the survey performed (6). Another is that this is the first study in Brazil and may have some influence on university curricula.

In summary, we have shown that identifying an emergency situation may not be a problem. Basic life support skills, however, were lacking in this population and were associated more with the career choice and personal interest of the individual than the existence of any formal training.

## Limitations

Our work has several limitations. Because we only dealt with new undergraduates, we cannot extrapolate our findings to the general population, but it would be reasonable to hypothesize that the situation may be worse. We tried to reach the whole population of undergraduates, so that the findings would be as representative as possible for internal consistency. Unfortunately, when we tried to compare with other references, there was no guide to sample size. We found papers with samples ranging from 100 to 3000, so we believe that our sample is representative. Furthermore, because there is a shortage of data about the State of São Paulo and Brazil, we believe that our study is original.

Another problem, as already mentioned, is the structure of the questionnaire. This could have limited the amount of information gathered, but this was an intentional choice, since we were trying to gather information in a brief questionnaire, so that its completion would not jeopardize the timeframe provided for the task. The open-ended question gave us a problem in extracting the content. We could perhaps have tried using another type of question, but we were afraid of biasing the answers by forcing a response from a set group of options, irrespective of the students’ knowledge.
